# Superantigenic Activation of Human Cardiac Mast Cells

**DOI:** 10.3390/ijms20081828

**Published:** 2019-04-12

**Authors:** Gilda Varricchi, Stefania Loffredo, Francesco Borriello, Antonio Pecoraro, Felice Rivellese, Arturo Genovese, Giuseppe Spadaro, Gianni Marone

**Affiliations:** 1 Department of Translational Medical Sciences, University of Naples Federico II, 80100 Naples, Italy; stefanialoffredo@hotmail.com (S.L.); francesco.borriello@childrens.harvard.edu (F.B.); anthonypek@msn.com (A.P.); argenove@unina.it (A.G.); spadaro@unina.it (G.S.); 2 Center for Basic and Clinical Immunology Research (CISI), 80100 Naples, Italy; 3 World Allergy Organization (WAO) Center of Excellence, 80100 Naples, Italy; 4 Division of Gastroenterology, Boston Children’s Hospital and Harvard Medical School, Boston, MA 02115, USA; 5 Centre for Experimental Medicine and Rheumatology, William Harvey Research Institute, Barts and The London School of Medicine and Dentistry, Queen Mary University of London, London E1 4NS, UK; rivelles@gmail.com; 6 Institute of Experimental Endocrinology and Oncology “Gaetano Salvatore”, National Research Council (CNR), 80100 Naples, Italy

**Keywords:** angiogenesis, heart, histamine, IgE, leukotriene C_4_, lymphangiogenesis, mast cells, myocardial infarction, prostaglandin D_2_, superantigens

## Abstract

B cell superantigens, also called immunoglobulin superantigens, bind to the variable regions of either the heavy or light chain of immunoglobulins mirroring the lymphocyte-activating properties of classical T cell superantigens. Protein A of *Staphylococcus aureus*, protein L of *Peptostreptococcus magnus*, and gp120 of HIV are typical immunoglobulin superantigens. Mast cells are immune cells expressing the high-affinity receptor for IgE (FcεRI) and are strategically located in the human heart, where they play a role in several cardiometabolic diseases. Here, we investigated whether immunoglobulin superantigens induced the activation of human heart mast cells (HHMCs). Protein A induced the de novo synthesis of cysteinyl leukotriene C_4_ (LTC_4_) from HHMCs through the interaction with IgE V_H_3^+^ bound to FcεRI. Protein L stimulated the production of prostaglandin D_2_ (PGD_2_) from HHMCs through the interaction with κ light chains of IgE. HIV glycoprotein gp120 induced the release of preformed (histamine) and de novo synthesized mediators, such as cysteinyl leukotriene C_4_ (LTC_4_), angiogenic (VEGF-A), and lymphangiogenic (VEGF-C) factors by interacting with the V_H_3 region of IgE. Collectively, our data indicate that bacterial and viral immunoglobulin superantigens can interact with different regions of IgE bound to FcεRI to induce the release of proinflammatory, angiogenic, and lymphangiogenic factors from human cardiac mast cells.

## 1. Introduction

The term “superantigen” (SAg) refers to several proteins synthesized by a variety of bacteria and viruses that not only mimic, but also exceed the activity of conventional antigens in activating T and B cells [1,2,3,4,5]. Typical antigens are processed by antigen-presenting cells (APCs) into small peptides that bind a distal groove in the molecules of the major histocompatibility complex (MHC) [6]. The peptide: MHC (p:MHC) complex on the APC surface acts as a ligand of both T cell receptor (TCR) α and TCR β variable domains on a few specific T cell clones. By contrast, SAgs bind directly to the lateral surfaces of the MHC class II molecules and to the Vβ domain of the TCR and thus bypass the processing and presentation of conventional antigens by APCs [7,8,9,10]. As a result, conventional antigens stimulate less than 1 in 10,000–100,000 T cells, while SAgs can stimulate up to 20% of all T cells [1,3]. A wide range of diseases from autoimmune and allergic disorders, neoplasia, and immunodeficiencies can be associated with SAgs [11,12,13,14,15].

In addition to classical T cell Sags, there are also B cell SAgs endowed with immunoglobulin (Ig)-binding capacity. In contrast to conventional antigens, which bind to both the heavy and light chain variable (V)-domains of Igs, B cell SAgs bind to the conserved sides of either the heavy (H)- or light (L)-chain [16,17,18], resulting in a massive proliferation of B cells. *Staphylococcus aureus* (*S. aureus*) is a source of several T cell SAgs (*S. aureus* enterotoxins: SE) [19]. Two staphylococcal B cell SAgs, *S. aureus* protein A and SEA, bind specifically to V_H_3 domain of human Igs, whereas SED, which is also a T cell SAg, binds to V_H_4 [11]. V_H_3 is the largest of human Ig germline V_H_ families; thereby, protein A can stimulate almost half of the B cells in the circulation [17]. Protein A is the archetypal B cell SAg and contains five homologous repeated domains, each of which can bind to all or most of the V_H_3^+^ Igs. *S. aureus* is a common pathogen causing toxic shock syndrome and endocarditis [20,21]. Most of clinical isolates of *S.aureus* synthesize protein A, which can be released from the cell wall [22]. Protein A has two binding sites for human Igs: the classical site binds Fcγ, a constant region of IgG [23] and an alternative site that binds the Fab portion of 15% to 50% of human polyclonal IgG, IgM, IgA, and IgE [24].

Similarly, glycoprotein 120 (gp120) of HIV-1 is a viral B cell SAg, because it interacts with Ig V_H_3^+^ [25,26]. The entry of HIV into host cells is mediated the interaction of viral glycoprotein [27] gp120 with CD4 [28] and chemokine receptors on the cell surface [29,30]. HIV gp120 is a member of the Ig SAg family [31,32,33]. Emergence of cardiovascular disease has become a leading concern for patients with HIV infection [34,35].

Protein L is a cell wall protein synthesized by *Peptostreptococcus magnus* (*P. magnus*) [36]. Protein L is a multi-domain protein that binds to some κ light chain variable domain without interfering with the antigen-binding site [37,38]. Protein L binds to the V domain of the κ light chains of Igs [39,40,41]. In particular, protein L binds with high affinity (~10^10^ M^−1^) only human V*k* I, V*k* III and V*k* IV subtypes, but does not interact with V*k* II subtype [42].

Mast cells are tissue resident immune cells present in most connective tissues including murine [43,44,45], canine [46,47], and human heart [48,49,50,51]. Mast cells are canonically considered key effectors of allergic responses [52,53,54,55,56] and are critical sentinels in immunity [57,58]. Mast cells and their mediators participate in a variety of pathophysiological processes including response to infections [58,59,60], angiogenesis [61,62,63,64,65], lymphangiogenesis [61,66], autoimmune disorders [67,68,69], cancer [70,71,72,73], and cardiometabolic diseases [49,74,75,76,77,78].

Human mast cells express the high-affinity receptor (FcεRI) for immunoglobulin E (IgE) and cross-linking of the IgE-FcεRI network induces the release of preformed (e.g., histamine, tryptase, chymase) and de novo synthesized lipid mediators (e.g., prostaglandin D_2_ (PGD_2_), cysteinyl leukotriene C_4_ (LTC_4_)). We have previously shown that several immune cells, such as human lung mast cells [61], basophils [79], macrophages [80,81], and neutrophils [82], produce angiogenic (e.g., vascular endothelial growth factor A:VEGF-A) and/or lymphangiogenic factors (e.g., vascular endothelial growth factor C: VEGF-C) [52,61,81]. However, there is a marked heterogeneity of human mast cells with respect to the mediators released from cells isolated from different anatomic sites [83,84,85].

This study has been undertaken to evaluate whether bacterial (protein A and protein L) and viral (gp120) superantigens induce the release of proinflammatory, angiogenic, and lymphangiogenic factors from human cardiac mast cells.

## 2. Results

### 2.1. Effect of Human IgG Anti-IgE on Mediator Release from HHMCs

**Figure 1:**
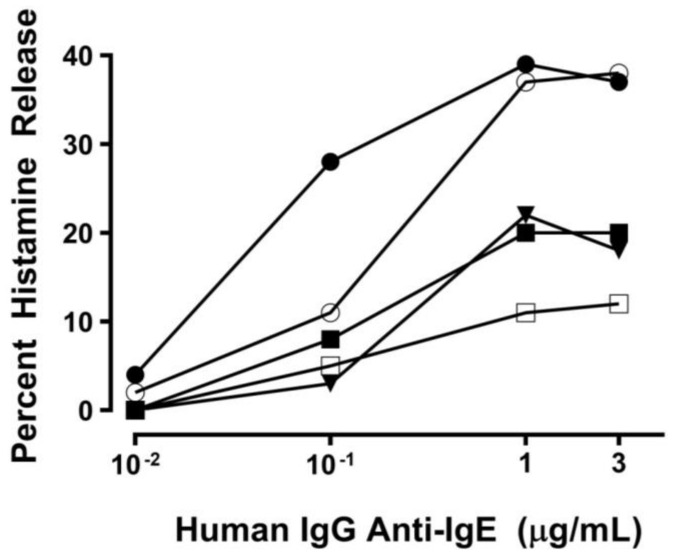
Effect of increasing concentrations of human IgG anti-IgE purified from the serum of a patient with atopic dermatitis [86] on histamine release from five different preparations of human heart mast cells (HHMCs). HHMCs were incubated (45 min at 37 °C) with the indicated concentrations of human IgG anti-IgE. Each point shows the mean of duplicate determinations. Each symbol represents the results from an individual donor.

We have previously reported that IgG anti-IgE purified from the serum of a small percentage of atopic dermatitis patients can induce histamine and LTC_4_ release from human basophils [86]. The activating property of human IgG anti-IgE (H-aIgE) is mediated by the interaction with membrane-bound IgE on human basophils. Therefore, we used this human autoantibody to activate human heart mast cells (HHMCs) in vitro. Figure 1 shows that H-aIgE (10^−2^ to 3 μg/mL) induced a concentration-dependent histamine release from five different preparations of HHMCs. Four preparations of IgG (10^−2^ to 3 μg/mL) purified from the serum of normal donors did not cause histamine release (data not shown). These results suggest that mast cells isolated from human heart express IgE bound to FcεRI.

**Figure 2:**
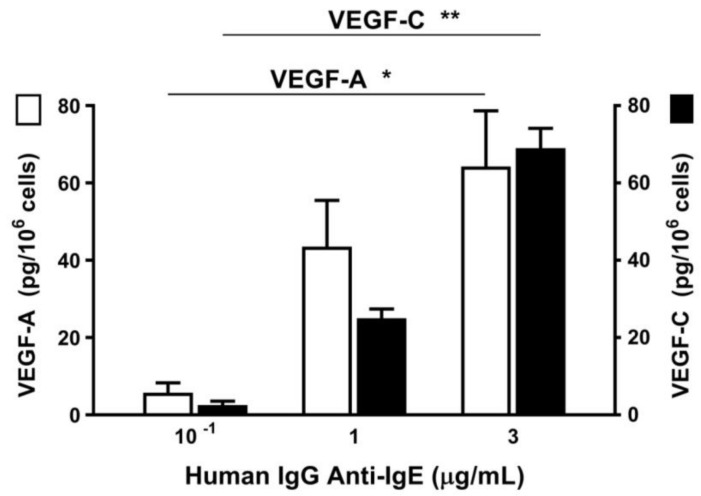
Effect of increasing concentrations of human IgG anti-IgE on the release of vascular endothelial growth factor-A (VEGF-A) and vascular endothelial growth factor-C (VEGF-C) from HHMCs from four donors. HHMCs were incubated (6 h at 37 °C) in the presence of the indicated concentrations of human IgG anti-IgE. Each bar is the mean ± SEM. * *p* < 0.05; ** *p* < 0.01.

Vascular endothelial growth factors (VEGFs) are involved in new vessel formation and play a central role in cardiac pathophysiology [87]. Therefore, we evaluated the release of angiogenic (VEGF-A) and lymphangiogenic factors (VEGF-C) induced by H-aIgE from HHMCs. Figure 2 shows that H-aIgE induced a concentration-dependent release of both VEGF-A and VEGF-C from four different preparations of HHMCs.

### 2.2. Effect of Bacterial Superantigens on Mediator Release from HHMCs

**Figure 3:**
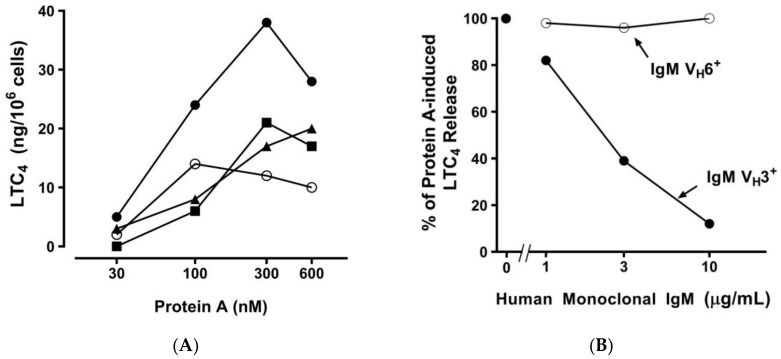
(**A**) Effect of increasing concentrations of protein A on the de novo synthesis of cysteinyl leukotriene C_4_ (LTC_4_) from four different preparations of HHMCs. HHMCs were incubated (45 min at 37 °C) with the indicated concentrations of protein A. Each point shows the mean of duplicate determinations. Each symbol represents the results from an individual donor. (**B**) Effect of preincubation of protein A with human monoclonal IgM on the activation of HHMCs. Protein A (300 nM) was preincubated (15 min at 37 °C) with increasing concentrations (1 to 10 μg/mL) of human monoclonal IgM V_H_3^+^ or IgM V_H_6^+^. HHMCs were then added and incubation continued for another 45 min at 37 °C. Each point shows the mean of duplicate determinations of a representative experiment. Similar results were obtained in two other experiments.

Figure 3A shows that protein A induced a concentration-dependent release of LTC_4_ from four different preparations of HHMC. To evaluate the mechanism by which protein A activates HHMCs, it was preincubated with human monoclonal IgM possessing different V_H_ domains. Figure 3B shows that human monoclonal IgM V_H_3^+^ dose-dependently inhibited the LTC_4_-releasing activity of protein A. By contrast, human monoclonal IgM V_H_6^+^ had no inhibitory effect. These findings are compatible with the hypothesis that protein A activates HHMCs through the binding to IgE V_H_3^+^ bound on FcεRI.

**Figure 4:**
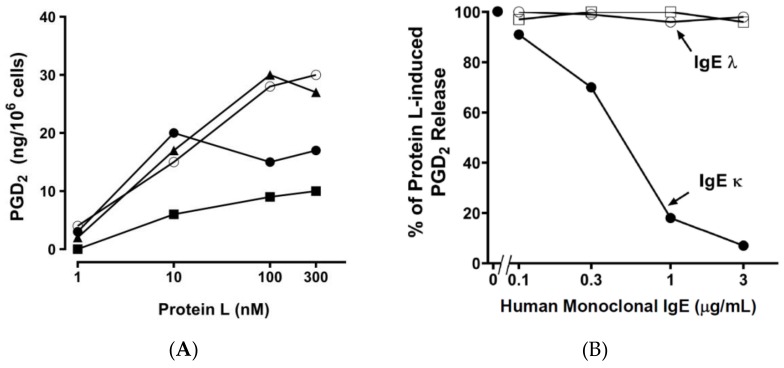
(**A**) Effect of increasing concentrations of protein L on the de novo synthesis of prostaglandin D_2_ (PGD_2_) from four different preparations of HHMCs. HHMCs were incubated (45 min at 37 °C) with the indicated concentrations of protein L. Each point shows the mean of duplicate determinations. Each symbol represents the results from an individual donor. (**B**) Effect of preincubation of protein L with human monoclonal IgE on the activation of HHMCs. Protein L (100 nM) was preincubated (15 min at 37 °C) with increasing concentrations (0.1 to 3 μg/mL) of two human monoclonal IgE λ light chain and one human monoclonal IgE κ light chain and incubation continued for another 45 min at 37 °C. Each point shows the mean of duplicate determinations of PGD_2_ of a representative experiment. Similar results were obtained in two other experiments.

We have previously found that *P. magnus* and protein L activate human basophils and mast cells [39,41]. Figure 4A shows that increasing concentrations of protein L induced de novo synthesis of PGD_2_ from HHMCs. The activating property of protein L (100 nM) was inhibited by preincubation with increasing concentrations (0.1 to 3 μg/mL) of human monoclonal IgE *k*, but not by two human monoclonal IgE λ (Figure 4B). These results are compatible with the hypothesis that protein L activates HHMCs through the interaction with the *k* light chain of IgE on cardiac mast cells.

### 2.3. Effect of Viral Superantigens on Mediator Release from HHMCs

**Figure 5:**
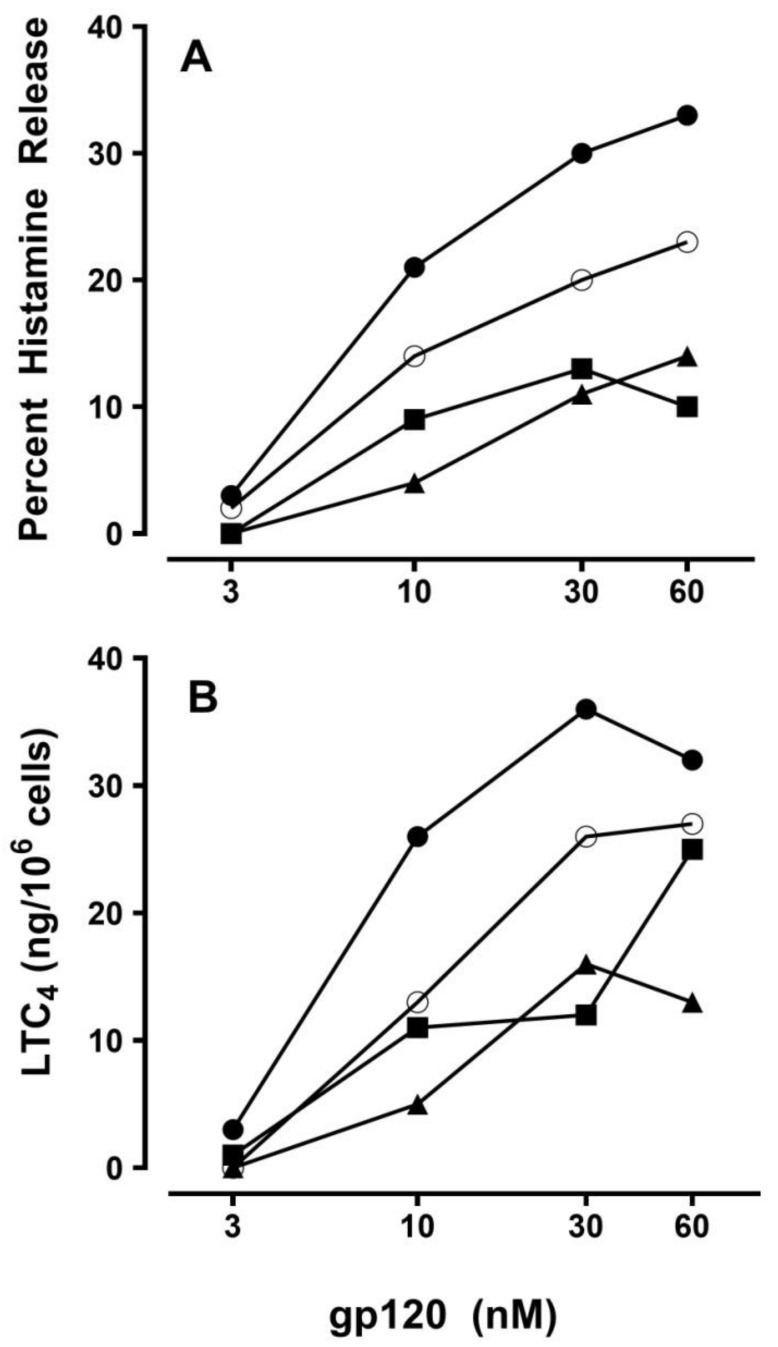
Effect of increasing concentrations of human immunodeficiency virus (HIV) gp120 on mediator release from four different preparations of HHMCs. HHMCs were incubated (45 min at 37 °C) with the indicated concentrations of gp120. At the end of incubation, the concentrations of histamine (**A**) and LTC_4_ (**B**) were measured in the four supernatants. Each point shows the mean of duplicate determinations. Each symbol represents the results from an individual donor.

Figure 5 shows the results of four independent experiments in which we incubated HHMCs with recombinant gp120. These experiments demonstrated that increasing concentrations of gp120 stimulated the release of histamine (Figure 5A) and the de novo synthesis of LTC_4_ from HHMCs (Figure 5B). Preincubation of gp120 (30 nM) with increasing concentrations (0.1 to 3 μg/mL) of human monoclonal IgE V_H_3^+^ inhibited the releasing activity of gp120 (data not shown). These results indicate that gp120 activates HHMCs by interacting with IgE V_H_3^+^ bound to FcεRI.

**Figure 6:**
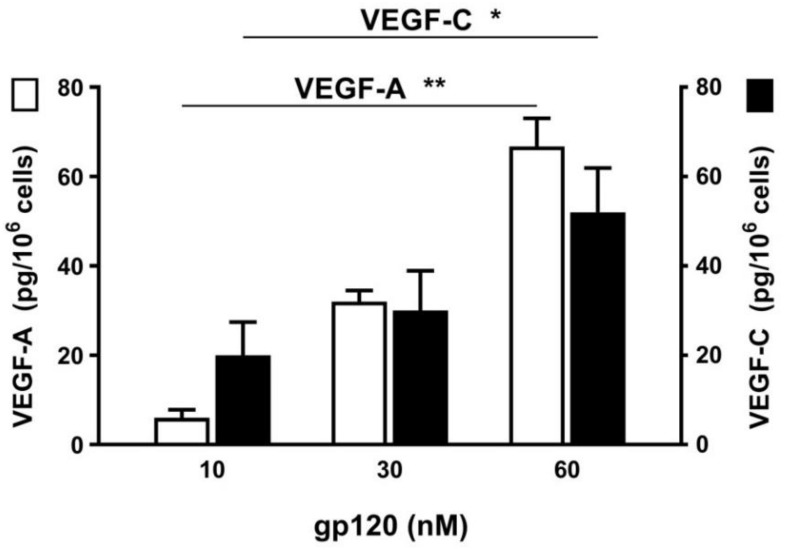
Effect of increasing concentrations of HIV gp120 on the release of VEGF-A (open bars) and VEGF-C (black bars) from four different preparations of HHMCs. HHMCs were incubated (6 h at 37 °C) in the presence of the indicated concentrations of gp120. Each bar shows the mean ± SEM. * *p* < 0.05; ** *p* < 0.01.

We then cultured HHMCs with increasing concentrations of recombinant gp120 (10 to 60 nM) for 6 h at 37 °C. At the end of this incubation the release of VEGF-A and VEGF-C was assayed in the supernatants of mast cells. Figure 6 shows the results of three preparations of HHMCs, indicating that gp120 induced the release of angiogenic (VEGF-A) and lymphangiogenic (VEGF-C) factors from HHMCs.

## 3. Discussion

This study shows that primary mast cells isolated from human myocardial tissue can be activated by a human IgG anti-IgE isolated from the serum of a patient with atopic dermatitis. These results are compatible with the hypothesis that HHMCs bind IgE, which has a role not only in allergic diseases [53,88] but also in several cardiovascular disorders [89,90,91]. Bacterial (protein A and protein L) and viral (gp120) superantigens can activate HHMCs to release a variety of proinflammatory (histamine, LTC_4_, PGD_2_), angiogenic (VEGF-A), and lymphangiogenic (VEGF-C) mediators. The releasing activity of protein A and gp120 appears to be mediated by interaction with the V_H_3 region of IgE on HHMCs. By contrast, protein L of *P. magnus* activates HHMCs by interaction with the κ light chains of IgE on cardiac mast cells. Our findings provide evidence, to our knowledge for the first time, that the immunologic (human IgG anti-IgE) and superantigenic activation of human myocardial mast cells can induce the release of angiogenic and lymphangiogenic factors.

Mast cells are present in strategically important locations of murine [43,92] and human heart [48,49,51,77]. Mast cells are present in atherosclerotic lesions [50,93] and promote atherogenesis [89]. These cells and their mediators are also involved in cardiometabolic diseases [78], myocardial infarction [76] and remodeling [94], atrial fibrillation [95], thromboembolism [45,51,96], and myocarditis [74,97,98]. Therefore, understanding how cardiac mast cells participate in these inflammatory disorders could help in the development of targeted therapies for these common diseases.

Serum IgE levels are elevated in patients with myocardial infarction [90,91] and coronary artery disease compared to controls [89]. Moreover, IgE and FcεRI are overexpressed in human atherosclerotic lesions. These findings suggest that mast cells and perhaps other immune cells expressing FcεRI (e.g., dendritic cells, macrophages, basophils, platelets) [89,99,100] could play a role in the pathogenesis of human atherosclerosis. Previous studies have demonstrated that autoantibodies anti-IgE and anti-FcεRI can occur in several immunologic disorders [86,101,102,103,104]. In this study we found that a human IgG anti-IgE induced the release of histamine, VEGF-A, and VEGF-C from HHMCs. To our knowledge this is the first evidence that cross-linking of IgE on human myocardial mast cells can induce the release of angiogenic factors. Angiogenesis, the process by which new capillaries develop from the pre-existing vasculature [105], plays a central role in cardiac pathophysiology [87,106]. VEGF-A is a pivotal mediator in angiogenesis and is synthesized by several immune cells [61,79,81,82,107,108,109,110]. The possibility that human cardiac mast cells can contribute to myocardial angiogenesis, a process of major relevance in cardiac pathophysiology [106], requires further investigations.

The mammalian heart is rich of lymphatic vessels [111,112] and their number is increased in human heart following myocardial infarction, in atherosclerosis lesions, and in endocarditis [113,114]. The involvement of VEGF-C in salt-sensitive hypertension [115,116] and in coronary artery development [117] further add to the implications of lymphangiogenic factors in cardiovascular diseases [112]. Our results provide the first indication to our knowledge that immunologic and superantigenic activation of HHMCs leads to the production of VEGF-C, a major selective mediator of lymphangiogenesis [112].

*S. aureus* is an important human pathogen implicated in sepsis and endocarditis [118], and sepsis is a risk factor for cardiac arrhythmias [119]. This study demonstrates that protein A induces the release of LTC_4_ from HHMCs through the interaction of the V_H_3 region of IgE. These results extend on the previous observation that protein A induces the in vitro release of histamine from HHMCs [40]. Recently, it has been reported that in vivo challenge with protein A resulted in fatal anaphylaxis involving V_H_3^+^ immunoglobulin interaction on mast cells and basophils [120]. Given the relevance of histamine and cysteinyl leukotrienes in heart pathophysiology [121,122,123], our results might explain, at least in part, how *S. aureus* can cause heart damage in patients with sepsis.

Protein L synthesized by *P. magnus* induces the de novo synthesis of PGD_2_ from HHMCs, by interacting with the κ light chains of IgE on HHMCs. These results extend previous findings indicating that protein L induces the release of preformed histamine from HHMC [40]. Therefore, protein L is a complete secretagogue capable of releasing preformed and de novo synthesized mediators implicated in cardiovascular pathophysiology [121,122,123,124].

Our results provide the first indication that HIV gp120 activates HHMCs, thus acting as Ig SAg. Previous studies from our group have shown that gp120 induces the release of cytokines (IL-4 and IL-13) from human basophils [26]. Collectively, these findings support the hypothesis that virus-bound or shed gp120 [125] can function as a viral superantigen activating HMMCs and basophils to release proinflammatory mediators (histamine, LTC_4_), cytokines (IL-4 and IL-13), and angiogenic/lymphangiogenic factors (VEGF-A and VEGF-C), thus contributing to the dysregulation of immune system in HIV infection. The successful rollout of anti-viral therapy ensured that HIV infection is managed as a chronic condition. Persistent inflammation and immune dysregulation associated with HIV leads to accelerated aging and cardiovascular diseases [34,35,126,127]. HIV-positive persons are, therefore, exhibiting increasing cardiovascular complications [34,35]. Our results, indicating that gp120 can induce the release of potent proinflammatory (histamine and LTC_4_) mediators that exert cardiovascular effects [121,122,123] from myocardial mast cells, might explain, at least in part, how HIV can cause heart damage.

In this study we have identified several immunological, bacterial, and viral products that activate human cardiac mast cells through the interaction with IgE bound to FcεRI. However, mast cells can be activated by non-IgE- mediated stimuli such as cytokines (e.g., IL-33, SCF) [65,77,128], TLR ligands [60,129], and neuropeptides [52,130]. Additional studies are necessary to evaluate the effects of non-IgE-mediated stimuli on the release of proinflammatory mediators, angiogenic and lymphangiogenic factors from human cardiac mast cells.

Our study has a limitation which has to be pointed out. It was performed using primary mast cells isolated from myocardial tissue obtained from patients undergoing heart transplantation. Thus, these mast cells might have different characteristics from cells obtained from healthy donors. We have previously had the opportunity to address this important issue by comparing the release of mediators from mast cells isolated from failing hearts and from subjects who died in accidents without cardiovascular diseases [77]. We found quantitative, but not qualitative differences in the release of mediators from “normal” cardiac mast cells when compared with those from explanted hearts.

In conclusion, our results demonstrate that bacterial and viral immunoglobulin superantigens can activate primary human cardiac mast cells to release vasoactive and proinflammatory mediators and angiogenic and lymphangiogenic factors.

## 4. Materials and Methods

### 4.1. Reagents

HClO_4_ (Baker Chemical Co., Deventer, The Netherlands), BSA, piperazine-N,N′-bis (2-ethanesulfonic acid), L-glutamine, antibiotic-antimycotic solution (10,000 IU penicillin, 10 mg/mL streptomycin, and 25 μg/mL amphotericin B), hyaluronidase, chymopapain, elastase type I, LTC_4_, and PGD_2_ (Sigma-Aldrich, St. Louis, MO, USA), collagenase (Worthington Biochemical Co., Freehold, NJ, USA), Hanks’ balanced salt solution and fetal calf serum (FCS) (GIBCO, Grand Island, NY, USA), deoxyribonuclease I and pronase (Calbiochem, La Jolla, CA, USA), RPMI 1640 with 25 mM HEPES buffer, Eagle’s minimum essential medium (Flow Laboratories, Irvine, UK), Percoll (Pharmacia Fine Chemicals, Uppsala, Sweden), (^3^H)-LCT_4_ and (^3^H)-PGD_2_ (New England Nuclear, Boston, MA, USA) were commercially purchased. CD117 MicroBead kit was purchased from Miltenyi Biotech (Bologna, Italy). The rabbit anti-LTC_4_ and anti-PGD_2_ antibodies were a gift of Dr. Lawrence M. Lichtenstein (The Johns Hopkins University, Baltimore, MD, USA). Human IgG anti-IgE was purified from the serum of a patient with atopic dermatitis as described elsewhere [86].

### 4.2. Buffers

The Pipes (P) buffer used in these experiments was a mixture of 25 mM Pipes, 110 mM NaCl, 5 mM KCl, pH 7.37, referred to as P. P2CG, contains, in addition to P, 2 mM CaCl_2_ and 1 g/L dextrose [49]; pH was titrated to 7.4 with sodium bicarbonate. PGMD contains 0.25 g/L MgCl_2_·6H_2_O, 10 mg/L DNase, and 1 g/L gelatin in addition to P, pH 7.37.

### 4.3. Human Monoclonal IgM and IgE and Human Polyclonal IgG

Monoclonal IgM were purified from the serum of patients with Waldenström’s macroglobulinemia as described elsewhere [77]. IgE myeloma proteins were purified from the serum of three patients described elsewhere [131,132]. Variable regions of these monoclonal IgM were determined using a panel of primary sequence-dependent V_H_ and V_K_ family specific reagents that identify framework regions [133]. Human polyclonal IgG were purified from the serum of healthy donors as described elsewhere [132].

### 4.4. Isolation of HHMCs

The study was approved by the Ethics Committee of the University of Naples Federico II (Protocol: Human MC No 7/19, 05/03/2009). The heart tissue was obtained from patients undergoing heart transplantation at the Deutsches Herzzentrum, Berlin, mostly for cardiomyopathy [77]. The explanted heart was immediately immersed in cold (4 °C) cardioplegic solution and was processed within 5 to 18 h of removal. Fat tissue, large vessels, and pericardium were removed. The tissue was finely minced into 2–5 mm fragments, suspended in P buffer (10 mL/g of wet tissue), and washed by centrifugation 3 times. After each centrifugation, the heart fragments were filtered through a 150 μm pore Nytex cloth (Tetko, Elmsford, NY, USA). Fragments were incubated (15 min, 37 °C) under constant stirring in P buffer containing 10 mg collagenase/g of wet tissue. At the end of the incubation the cell suspension was filtered through a 150 μm pore Nytex cloth and three additional cycles of enzymatic digestion were performed. After the last procedure, the cells were centrifuged (150× *g*, 22 °C, 8 min) and filtered through a 60 μm pore Nytex cloth to remove large particles and large cells (mostly myocytes). Lastly, cells were washed twice in PGMD by centrifugation (150× *g*, 22 °C, 8 min). Cell pellets were resuspended in P buffer containing 2% BSA and centrifuged (25× *g*, 22 °C, 2 min) to remove sedimented myocytes (>100 μm long). Supernatants containing endothelial cells, fibroblasts, and mast cells were then collected and centrifuged (150× *g*, 22 °C, 8 min). HHMC were partially purified by flotation through a discontinuous Percoll gradient [77]. The purity of these populations ranged from 0.1% to 18%. The enzymatic dispersion tissue yields ≈ 5 × 10^4^ mast cells per gram of heart tissue. HHMCs were further purified using a CD117 MicroBead kit sorting system (Miltenyi Biotec, Bologna, Italy). Mast cell purities using these techniques ranged from 26% to 58% and was assessed by toluidine blue staining.

### 4.5. Histamine Release Assay

HHMCs (≈ 3 × 10^4^ mast cells per tube) were resuspended in P2CG, and 0.3 mL of the cell suspension were placed in 12 × 75 mm polyethylene tubes and warmed to 37 °C; 0.2 mL of each prewarmed releasing stimulus was added, and incubation was continued at 37 °C for 45 min [39]. The reaction was stopped by centrifugation (1000× *g*, 22 °C, 2 min), and the supernatants were stored at −80 °C for subsequent assay of histamine, LTC_4_, and PGD_2_ content. The cell-free supernatants were assayed for histamine with an automated fluorometric technique [134]. To calculate histamine release as a percentage of total cellular histamine, the “spontaneous” release from mast cells was subtracted from both numerator and denominator. All values are based on means of duplicate determinations which differed by less than 10%.

### 4.6. Immunoassay of LTC_4_ and PGD_2_

LTC_4_ and PGD_2_ were measured in duplicate determinations by radioimmunoassay [39,135]. The anti-LTC_4_ and anti-PGD_2_ antibodies are highly selective, with less than 1% cross-reactivity to other eicosanoids [39,135].

### 4.7. VEGF-A and VEGF-C Release

HHMCs (≈ 4 × 10^4^ mast cells/per tube) were incubated (37 °C, 6 h) in RPMI 1640 containing 5% FCS, 2 mM L-glutamine, and 1% antibiotic-antimycotic solution, and activated with various concentrations of stimuli. At the end of incubation, cells were centrifuged (1000× *g*, 4 °C, 5 min) and the supernatants were stored at −80 °C for subsequent determination of mediator release. VEGF-A and VEGF-C were measured in duplicate determinations using commercially available ELISA kits (R&D System, Minneapolis, MN, USA) as previously described [136]. The ELISA sensitivity is 31.1–2000 pg/mL for VEGF-A and 62–4000 pg/mL for VEGF-C.

### 4.8. Statistical Analysis

Values were expressed as means ± SEM (standard error of the mean). The one-way repeated measures analysis of variance (ANOVA) with Greenhouse–Geisser corrections was used to examine the variations of continuous variables at different experimental conditions. Results were analyzed with GraphPad Prism software (version 8.01; GraphPad Software, La Jolla, CA, USA), and *p* values of less than 0.05 were considered significant.

## Abbreviations

*S. aureus*	*Staphylococcus aureus*
*P. magnus*	*Peptostreptococcus magnus*
LTC_4_	Cysteinyl leukotriene C_4_
HHMCs	Human heart mast cells
Ig	Immunoglobulin
FcεRI	High-affinity receptor for IgE
PGD_2_	Prostaglandin D_2_
gp120	Glycoprotein 120
VEGF	Vascular endothelial growth factor
SAg	Superantigen
APCs	Antigen-presenting cells
MHC	Major histocompatibility complex
TCR	T cell receptor
V	Variable
H	Heavy
L	Light
SE	*Staphylococcus aureus* enterotoxins
IL	Interleukin
HIV	Human immunodeficiency virus
FCS	Fetal calf serum
BSA	Bovine serum albumin
H-aIgE	Human IgG anti-IgE
